# Quantitative phase imaging endoscopy with a metalens

**DOI:** 10.1038/s41377-024-01587-y

**Published:** 2024-11-08

**Authors:** Aamod Shanker, Johannes E. Fröch, Saswata Mukherjee, Maksym Zhelyeznyakov, Steven L. Brunton, Eric J. Seibel, Arka Majumdar

**Affiliations:** 1 Department of Electrical and Computer Engineering, University of Washington, Seattle, WA 98195 USA; 2 Center for Vision Science, University of Rochester, New York, NY 14623 USA; 3 Department of Mechanical Engineering, University of Washington, Seattle, WA 98105 USA; 4 Department of Physics, University of Washington, Seattle, 98195 USA

**Keywords:** Imaging and sensing, Microscopy, Biophotonics, Micro-optics

## Abstract

Quantitative phase imaging (QPI) recovers the exact wavefront of light from intensity measurements. Topographical and optical density maps of translucent microscopic bodies can be extracted from these quantified phase shifts. We demonstrate quantitative phase imaging at the tip of a coherent fiber bundle using chromatic aberrations inherent in a silicon nitride hyperboloid metalens. Our method leverages spectral multiplexing to recover phase from multiple defocus planes in a single capture using a color camera. Our 0.5 mm aperture metalens shows robust quantitative phase imaging capability with a ${28}^{\circ}$ field of view and 0.${2}{\pi}$ phase resolution ( ~ 0.${1}{\lambda}$ in air) for experiments with an endoscopic fiber bundle. Since the spectral functionality is encoded directly in the imaging lens, the metalens acts both as a focusing element and a spectral filter. The use of a simple computational backend will enable real-time operation. Key limitations in the adoption of phase imaging methods for endoscopy such as multiple acquisition, interferometric alignment or mechanical scanning are completely mitigated in the reported metalens based QPI.

## Abstract (web summary)

Metalens with typical chromatic aberrations, when attached to the distal tip of a conventional endoscope, simultaneously encodes different depths into the RGB channels of a camera at the proximal end.

## Graphical abstract

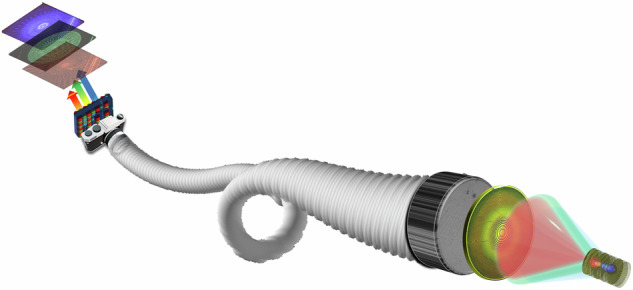

## Introduction

Thanks to the progress in nanofabrication technology, the availability of advanced electromagnetic simulators, and the ubiquitous presence of computing elements, imaging systems have been drastically miniaturized. On the one hand, it is now possible to create sub-wavelength flat diffractive optics, commonly known as meta-optics, with large phase gradients^1–4^. On the other hand, sophisticated computational imaging techniques can process the captured data to extract new information without adding significant hardware footprint^5,6^. Such miniaturization of imaging systems and sensors goes hand-in-hand with emerging applications, such as autonomous navigation systems and the Internet of things^7^. Biomedical imaging systems such as endoscopes^8,9^ or angioscopes that function as in-vivo probes also necessitate continuous miniaturization of optical elements. Imaging systems at ever-diminishing scales can benefit from capturing multi-dimensional data, often requiring information beyond conventional absorption or intensity-based measurements. Specifically, phase information is often needed for semi-transparent biomedical tissues.

Quantitative phase imaging (QPI) microscopy^10^ is able to extract precise quantitative information about optical pathlength-induced wavefront distortions from pure intensity measurements^11^. Knowledge and control of both the intensity and phase allow for surface metrology, diffraction tomography, and digital volumetric reconstruction of coherent light passing through tissue, surfaces, or translucent media. Conversely, controlling the phase enables beam shaping with holographic, diffractive, or meta-optical elements. These miniature phase plates are becoming an integral part of various emerging applications such as cameras and displays^12^, laser-based manufacturing, and light detection and ranging (LIDAR) systems. Conventional QPI relies on interferometric architectures with a reference beam that interferes with the scattered beam to encode the phase into intensity^10,13–16^. Coherence and alignment requirements make interferometric methods cumbersome for miniaturization. Hence, propagation-based phase-imaging methods^17^ are preferred in miniature imaging systems due to their self-referential and non-interferometric architecture, tolerance of partial coherence as well and ease of adoption in any imaging systems with a tunable focus.

Apart from biomedical endoscopy, applications of defocus-based QPI have vastly expanded since it was first proposed as a non-interferometric phase imaging method that was effective in partially coherent illumination conditions^18,19^. The more recent adoption of non-interferometric QPI includes X-ray phase imaging^20,21^, atomic and molecular structure imaging^22^, semiconductor lithography, and metrology^23^. Within the context of biomedical imaging, the ability to extract morphological information from the quantitative phase without contrast agents, biomarkers, or fluorescent labels enables the characterization of cell structure and function in its native environment^14,16^. QPI reconstructs wavefront shifts accumulated when light passes through tissues and cells with thickness or refractive index variations corresponding to their underlying morphology and optical density. While ex-vivo QPI for biomedical imaging has been widely adopted, in-vivo translation has been limited due to double pass geometry inherent in reflection mode (epi) imaging. Typical in-vivo imaging architectures rely on point scanning confocal systems along with spatial, temporal, or coherence gating (such as with a confocal pinhole at the detector or using a two-photon/swept spectrum source) to amplify signal from translucent tissue boundaries while rejecting out of focus light. Examples of successful in-vivo imaging systems include optical coherence tomography^24^, scanning laser ophthalmoscopy^25^, and scanning fiber endoscopy^26^. For both scanning and flood systems operating in reflection mode, phase information is scrambled due to double pass through the 3D tissue. However, phase shifts gathered during a single pass through the tissue can still be captured—by imaging at a deeper opaque layer^27^, with oblique illumination where the illumination and imaging paths are separated by a large angle of incidence^28,29^, or possibly by backlighting with a confined fluorescence target^30,31^. In either strategy, the ability to demonstrate QPI in transmission mode will directly enable the adoption of in-vivo imaging systems.

Additionally, most QPI strategies for in-vivo biomedical imaging employ interferometric methods^14,16,32^. Interferometric systems are cumbersome and expensive for in-vivo applications at ophthalmological (1–20 mm) or endoscopic (<2 mm) scales due to fine alignment requirements in interference-based measurements between the reference beam and scattered beam. Interferometric phase imaging systems also have strict coherence requirements, often necessitating the use of expensive tunable laser systems in addition to the scanner cost and alignment, for example, in optical coherence tomography (OCT). Alternatives to interferometric systems, such as lens-less or speckle-based adoptions of endoscopic phase imaging, have a lower hardware footprint, yet they suffer from speckle and honeycomb artifacts or calibration sensitivity where fiber bending ruins the reconstruction^33,34^. Here we address the challenges that hinder commercialization of QPI for clinical diagnosis due to high hardware complexity and cost, computational overhead and latency, or misalignment sensitivity requiring sophisticated manufacturing and packaging.

In this paper, we demonstrate a single-shot QPI method that relies on the inherent chromatic behavior of hyperboloid metalens. We demonstrate a video rate imaging modality that can add phase imaging capability to most existing endoscopy platforms with a white light source, without any additional hardware or significant computational cost. Metalenses suffer from strong axial chromatic aberration, with a wavelength-dependent focal length following a relation: $\delta f/f=-\delta \lambda /\lambda$^35^. In recent years, researchers have developed many techniques to mitigate the chromatic aberrations in metalens^3,5,6,36^. Here, we instead harness this chromatic aberration for QPI, especially in the context of endoscopic applications where the phase is deterministically distorted on passage through fiber cores. Various recent publications have demonstrated three-dimensional imaging using meta-optics^37,38^, yet quantitative phase retrieval through a coherent fiber bundle using meta-optics has not yet been demonstrated. The demonstrated QPI using sophisticated meta-optical designs is unsuitable for fiber endoscopy^39^ and often needs elaborate end-to-end phase calibration for each fiber in a fiber bundle^40^. Our single-shot QPI approach overcomes alignment or acquisition limitations of typical phase imaging methods by extracting the whole electric field from a single bright-field color image. We demonstrate robust phase retrieval with commercial-off-the-shelf light-emitting diodes (LED) and color cameras at real-time acquisition speeds. We further demonstrate the ability to decipher structural features from spectral information in meta-optical endoscopy. Since our meta-optic encodes phase into intensity before transmitting through the endoscope, our method has a high potential for translational impact when imaging through an optical fiber or other media that preserves spectral intensity $I({\lambda }_{{\rm {r}}},{\lambda }_{{\rm {g}}},{\lambda }_{{\rm {b}}})$ while deterministically distorting or even completely scrambling coherent phase $\phi$.

## Results

Harnessing the focal length dispersion in a metalens, we have developed a volumetric imaging technique that encodes depth into spectrum. The method is non-interferometric since the phase is computed from diverse defocus measurements; multiple focal planes are encoded in the RGB color channels in a simultaneous acquisition. The red, green, and blue channels are centered around 625, 530, and 455 nm, respectively, the central wavelength determined both by the spectral response of the Bayer filter in the CCD camera and the spectrum of the white light LED excitation (see the “Methods” section)^41^.

### QPI with the transport of intensity equation

A partially coherent light beam passing through transparent scatterers encodes nanometer scale height or refractive index variations into the phase of the wavefront as $\phi =\frac{2\pi }{\lambda }\times h\times \delta n$, where *h* is the sample height and $\delta n$ is the refractive index difference between the sample and the surrounding media ($\delta n \sim 0.5$ for glass in air). The effective phase gathered during propagation through the sample is encoded into the complex-valued, scalar electric field $\tilde{E}=\sqrt{I(x,y)}{{\rm {e}}}^{i\phi (x,y)}$, where $I(x,y)$ is the 2D intensity that can be measured on a CCD or photodiode. For a linear, time-invariant system, the electric field after propagation through a distance $z$ (where ${z}^{2}\gg {x}^{2}+{y}^{2}$) is given by the convolution of the input field with the paraxial Green’s function or point spread function (PSF) as: ${\tilde{E_{z}}}\left(x,y\right)=\tilde{E}\left(x,y\right)* \frac{{{\rm {e}}}^{{ikz}}}{i\lambda z}{{\rm {e}}}^{i\frac{2\pi }{\lambda }\frac{{x}^{2}+{y}^{2}}{2z}}$ (derivation in S1). A further approximation that assumes propagation to be perturbations along one principle direction ($k\approx {k}_{z})$ yields two complementary equations^32,33^ as follows.

Transport of intensity equation (TIE):

$$\frac{{{\rm {d}}I}}{{{\rm {d}}z}}=-\frac{\lambda }{2\pi }\vec{\nabla }.I\vec{\nabla }\phi$$

Transport of phase equation (TPE):

$$\frac{2\pi }{\lambda }\frac{{\rm {d}}\phi }{{{\rm {d}}z}}=-\vec{\nabla }\phi .\vec{\nabla }\phi +\frac{{\nabla }^{2}I}{2I}-\frac{{\left(\vec{\nabla }I\right)}^{2}}{4{I}^{2}}$$

where $\vec{\nabla }=\frac{{\rm {d}}}{{{\rm {d}}x}}\vec{x}+\frac{{\rm {d}}}{{{\rm {d}}y}}\vec{y}$ is the *in-plane* gradient normal to the propagation direction (z). Together, these two equations completely describe the coherent propagation of light as an alternating update between phase and intensity at each defocus step. The Transport of Intensity Equation is similar to the continuity equation for fluids, with light intensity substituting for fluid density. The Transport of Phase equation, on the other hand, is analogous to the momentum transport equation for fluids, where the time evolution of the fluid velocity is substituted by gradients of the light phase (see S1). A quantitative estimate of the phase can be solved from just the TIE, by using at least two intensity measurements (${{\rm {d}}I}$) at two propagation planes $({\rm {d}}z)$ to estimate the left-hand side of the TIE. Knowing the wavelength $(\lambda )$ and the intensity at the central plane $(I)$, the right-hand side is then solved for $\phi$ as a second-order differential equation (see the “Methods” section).

While the conventional propagation-based TIE assumes a fixed $\lambda$ and varying $z$, for a spectrally diverse measurement, we can swap the underlying variables in the assumption instead. Hence, for phase retrieval from differences in spectral intensity (d*I*/d*λ*) assuming a fixed defocus distance *z*, we rewrite the Transport of Intensity by swapping $\lambda$ and *z* as $\frac{{{\rm {d}}I}}{{\rm {d}}\lambda }=-\frac{z}{2\pi }\vec{\nabla }.I\vec{\nabla }\phi$^42^. Reframing the intensity change as a function of chromatic wavelength (d*I*/d*λ*) at constant defocus distance z describes the chromatic dispersion of the diffracted light at a certain propagation distance after scattering off an object with phase shift $\phi (x,y)$ and absorption $I(x,y)$. Hence, axial propagation $(z)$ is equivalent to spectral dispersion ($\lambda )$ according to the transport equation for intensity, which effectively treats the variable $\zeta =\lambda z$ as a single combined variable (validated experimentally to show $\zeta ={{\rm {constant}}}$ in Supplement S5).

#### Metalens design

**Fig. 1:**
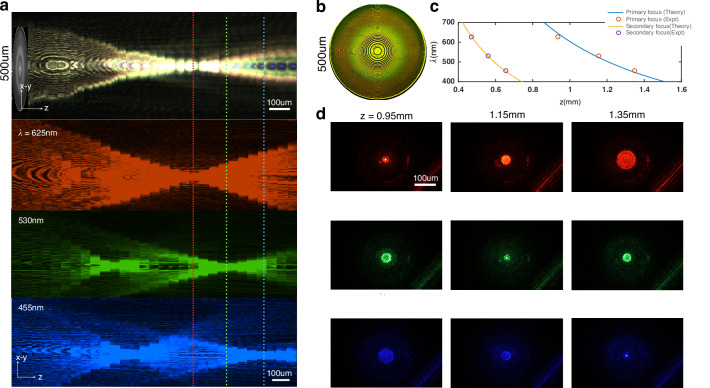
Metalens spectral characterization. **a** The metalens is illuminated with collimated light from an LED source with wavelengths centered at red (625 nm), green (530 nm), and blue (455 nm), respectively. Columns through the center of the measured camera intensity (*x*–*y*) are stacked through focus to create the longitudinal view (*y*–*z*) showing spectral dependence of focal length. The meta-optic is designed to focus green light at ~1 mm. The red light reaches focus first at 0.95 mm (top row), followed by green at *z* = 1.15 mm (middle row), and blue at *z* = 1.35 mm (bottom row). A weak secondary focus is seen at 0.5 mm for blue and green light. **b** Brightfield image of the fabricated hyperboloid lens surface with a ×10 microscope. **c** The focal length along the propagation direction ($$z)$$ as a function of wavelength $$(\lambda )$$ shows almost perfect fit to $$\lambda \propto \pi {r}^{2}/z$$. **d** The 2D PSF as a function of the focal distances for the RGB LEDs measured with a ×20 objective on a standard microscope

We now describe how a rudimentary metalens design preserves the conjugacy between focal distance and wavelength (Fig. 1). The metalens we use throughout this work is a simple hyperboloid lens. The intrinsic chromatic behavior of the metalens is akin to a spectral diffraction grating along the longitudinal propagation direction *z*, effectively having linear longitudinal chromatic aberration (LCA). In principle, our TIE-based phase retrieval method can also be applied to achromatic meta-lenses with a parabolic LCA by averaging the red and blue channels and subtracting the green to estimate the intensity derivative^42^. The hyperboloid lens with a phase profile along its radius $r$ as $\phi (r)=\frac{2\pi }{\lambda }(\sqrt{{r}^{2}+{f}^{2}}-f)\approx \frac{2\pi }{\lambda }\left(\frac{{r}^{2}}{2f}\right)$^43^, is designed to focus collimated green light ($\lambda =$530 nm) at a fixed focal distance $f$. With a white light source, the red (625 nm) and blue (455 nm) channels automatically focus behind and ahead of the focal plane for green, respectively (Fig. 1a, d). The focal shift between red and green $(\delta \lambda =95\,{\rm {{nm}}},\lambda =530\,{\rm {{nm}}},\,f\,=\,1\,{{\rm{mm}}})$ is calculated to be $\delta f=190\,{{\upmu m}}$ since $\lambda \delta f=-f\delta \lambda$. The measured $\lambda f$ values are nearly identical for the three colors, where *f* is the focal distance for the given $\lambda$ (see S5). We also measure the change in focal distance between the color channels $\delta f=180\,{{\upmu m}}$, which is almost identical to the predicted value (Fig. 1d).

We choose a nominal focal length of 1 mm for the 0.5 mm aperture hyperboloid metalens, with an effective numerical aperture (NA) of ~ $0.25$. The objective used to relay the metalens image has a larger NA of 0.75, hence being able to capture the entire angular bandwidth from the metalens. The metalens is made of thin film Si_3_N_4_ pillars on quartz. The appropriate pillar dimensions are computed by rigorous coupled wave analysis (see Supplement S5). Standard e-beam lithography followed by etching is used to fabricate the meta-optics. We utilize three color channels in a camera’s Bayer filter instead of taking three defocus measurements on a mechanical stage. The phase of the wavefront can be computed by inverting the previously introduced TIE that relates phase to defocus $z$ and, hence, to color $\lambda$^44^. Theoretically, the TIE is a linear second-order differential equation that needs only two measurements to determine two unknowns—intensity and phase. However, three measurements are preferred for a more accurate gradient measurement dI/d*λ*. In our application, where we rely on spectral aberrations to extract depth information, we will often only utilize two color channels (typically red or green) due to aberrations or magnification disparities in the blue channel. Next, we calibrate the spectral channels to find the equivalent defocus distances to achieve QPI by solving the linearized transport equations for partially coherent light.

#### Calibration of spectral vs. focal distances

**Fig. 2:**
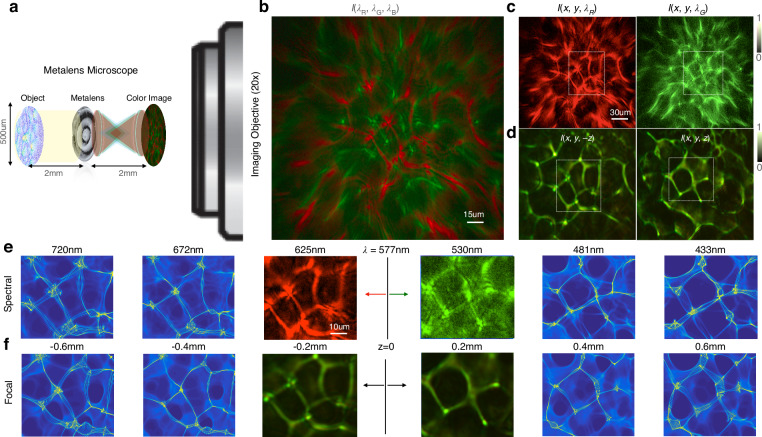
Spectral focal equivalence. **a** The metalens microscopy setup in imaging condition with the ×20 microscope objective (drawn to scale). The metalens is 2*f* (2 mm) away from the phase target, in this case, a precision diffuser, with an inverted image formed at 2*f* from the metalens. The microscope objective magnifies the real image by ×20 after passing through a tube lens onto a color CMOS camera. **b** The single shot color image captured on the camera. **c** Red and green simultaneously capture positive and negative defocus images on either side of the diffuser. **d** Equivalent positive and negative defocus measurements demonstrate that a *δλ* = 95 nm spectral shift (${\lambda }_{{\rm {R}}}=625\,{\rm {nm}}$, *λ*_G_ = 530 nm) corresponds to a *δf* = 190 μm focal length shift as shown previously, and hence to a ~0.4 mm shift in the imaging plane located 2*f* from the lens (since 2$\delta f=\pm 380\,{{\upmu m}}$). **e** Phase retrieval enables digital propagation of the electric field to extrapolate the image formed by wavelengths separated by ~48 nm steps over the entire visible spectrum. **f** Defocus images measured experimentally are also digitally propagated to show similar caustic patterns to those extrapolated numerically by propagating the spectral measurements

Our spectral QPI is first calibrated using a precision diffuser as a designer-disordered media (see the “Methods” section). The diffuser is chosen as a strong stochastic scatterer because it maximizes the propagation-based phase contrast. Once we have measured the dependence of spectral channels on focal distance (${\rm {d}}\lambda /{{\rm {d}}z}$), we solve the phase using the TIE by treating each color as an equivalent defocus measurement. Knowing the refractive index of the scatterer relative to the surrounding media ($\delta n=0.52$), the height (*h*) can be recovered from the solved quantitative phase ($\phi$). The metalens microscopy setup for calibration is shown in Fig. 2a. Collimated light from a white LED is incident on the planar, pure-phase diffuser. The diffuser is placed two focal lengths in front of the metalens, forming an image two focal lengths behind the metalens with unity magnification (Supplement S10). The microscope objective is placed with its working distance at the plane where the image is formed by the metalens. Since the target is a phase target, it is perfectly transparent at focus, with $I(x,y)=1$ (ground truth amplitude and phase shown in Supplement S7 and S8). The microscope objective relays the image formed by the metalens to a standard RGB color camera (see “Methods” section and S2). Positive and negative defocus give us strong caustics, similar to the bright focal lines in the bottom of a wavy pool. These caustics have complementary patterns on either side of the focus (Fig. 2b–d). Supplement S3 shows a continuous transformation between caustics formed with and without the metalens in place, highlighting the aberrations in the metalens as a deformation of caustic patterns. Caustics first appear at a defocus distance of about $\pm 100\,{{\upmu m}}$ estimated by the Talbot distance $\delta z=2{p}^{2}/\lambda$^45^, where *p* is the nominal feature size (about $7\,{{\upmu m}}$ for the diffuser) and $\lambda$ = 530 nm is the nominal wavelength. Red and green channels are shown to capture complementary caustic patterns, corresponding to positive and negative defocus measurements. By calibrating with defocus measurements of the diffuser, we find that the red and green channels correspond to two defocus planes separated by ${{\rm {d}}z}=190\,{{\upmu m}}$, as expected from the focal length measurements in the previous section. We are thus able to utilize two color channels as an effective through-focus measurement to compute the quantitative phase and, hence, the entire light field. The digital holographic reconstruction or refocusing is performed by convolution with the defocus PSF. Two color channels are sufficient to compute the phase based on a linear approximation for the derivative d*I*/d*λ*, although three channels give a better estimate, as explained earlier. Here, we mostly rely on the red and green channels for the phase retrieval since the blue channel has a much lower signal-to-noise ratio as well as more pronounced higher-order aberrations, especially near the phase resolution limit.

#### Quantitative phase microscopy

Next, we image the quantitative phase using the camera’s color channels in a single shot. We use the same broadband LED source, scattered by a known target and imaged through the metalens, then relayed by a microsope objective or endoscope fiber bundle onto the color (Bayer) filters of the camera. The single metalens-based imaging system is set up in a 4*f* imaging configuration in order to minimize the enclosing volume, with object distance and image distance being equal to twice the focal length (see S10).

**Fig. 3:**
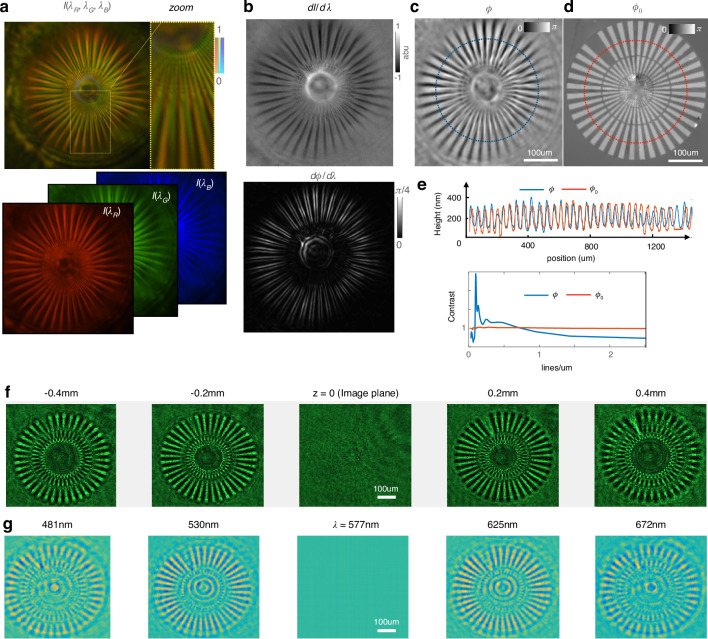
Quantitative phase imaging using a metalens for a 250 nm tall Siemens star target with refractive index 1.52. **a** Color image captured by a camera (top) with an integrated color filter gives RGB images in three separate channels (bottom). **b** The intensity difference between the color channels $({\rm{dI}}/{\rm{d}}{\rm{\lambda }})$ gives effective intensity change vs. color (top), while the phase change with color ${\rm{d}}{\rm{\phi }}/{\rm{d}}{\rm{\lambda }}$ is computed post-QPI (bottom) to reveal the spatial phase gradients as ${\rm{d}}{\rm{\phi }}/{\rm{d}}{\rm{\lambda }}\propto \vec{\nabla }{\rm{\phi }}.\vec{\nabla }{\rm{\phi }}$. **c** The recovered QPI image gives the exact height measurement of the test target. **d** The ground truth is measured on a Nikon microscope with a commercial phase imaging camera. **e** The recovered phase (blue) is compared with the ground truth (red) along the cutlines in **c** and **d** after converting to equivalent height value as ${h}=\frac{{\rm{\phi }}}{\Delta {n}}\frac{{\lambda }}{2{\pi }}$ (top). The QPI contrast as a function of spatial frequency calculated at different distance from the center shows <1 µm lateral resolution where the contrast dips below unity (bottom). **f** Experimentally measured intensity images of the Siemens star target as a function of defocus for 200 µm defocus steps. **g** Computational reconstruction of the spectral stack from a single color image measurement when compared with experimentally measured defocus images in **f** proves the accuracy of our phase retrieval. Notice how the bright spoke/dark gap flip contrast to dark spoke/bright gap on either side of focus, in both measurement and simulation

For a ~25 mW white light LED source, the color camera integration time for our single shot acquisition is <90 ms, which enables phase imaging at >10 Hz video rate. Since we retrieve the phase information from the simultaneous acquisition of three colors using the in-built Bayer filter in the color camera, the phase frame rate is the same as the intensity frame rate. The color camera, however, loses ×3 spatial resolution compared to a monochromatic array sensor since the total number of pixels is divided among three color channels. However, with the ×20 objective magnification onto the camera plane, the diffraction-limited spot size (1 µm × 20) is sufficiently oversampled by the color camera pixel size of 3.2 µm by a factor of ~6.25. Using a spectrometer, we estimate the effective peak wavelengths to be 625, 530, and 455 nm as the peaks of the overlap between the detector spectral sensitivity and the source emission spectrum (see the “Methods” section). We then use the intensity gradients vs wavelength (d*I*/d*λ*) in the TIE to compute the quantitative phase *ϕ*^44^; subsequently, we validate our calculated phase against the measured ground truth (Fig. 3b–d). A Siemens star phase target (Benchmark Inc.™) with a refractive index of 1.52 ($\delta n=0.52)$ is used to validate our method. The Siemens star rotates in opposite directions on either side of the focus (see S4). Once the phase has been solved, we validate that the phase derivative as a function of the wavelength highlights the edges of the star target where the spatial phase gradients are the strongest (Fig. 3b, bottom). This edge-enhanced contrast is also predicted by the Transport of Phase Equation as ${\rm {d}}\phi /{\rm {d}}\lambda \propto \vec{\nabla }\phi .\vec{\nabla }\phi$. Subsequently, the retrieved phase is shown to be accurate by two methods—first by measuring the Siemens star target with a commercial phase imaging camera for the ground truth (see Supplement S7 and S8 for SEM images and optical ground truth of the Siemens star); and second, by digitally propagating the recovered electric field and comparing with measured defocused images (Fig. 3f, g).

#### Noise and resolution

Our phase retrieval method shows $0.2\pi$ resolution in the vacuum of the retrieved phase. For the ex-vivo experiments in etched glass (with index contrast $\delta n=0.52$) the corresponding height sensitivity is $\delta h=\frac{0.2\pi }{2\pi }\left(\frac{\lambda }{0.52}\right)=$ 0.19$\lambda \approx 100\,{\rm {{nm}}}$ with green light at $\lambda =530\,{\rm {{nm}}}$. The 100 nm phase resolution is verified experimentally, limited only by the noise in the measured intensity derivative ${{\rm {d}}I}/{\rm {d}}\lambda$ (Supplement S9). Additionally, since we take differences between the color channels to estimate the intensity derivative, the background blur common to the spectral channels due to multiple scattering is eliminated, similar to other low-coherence optical sectioning techniques such as OCT^24^ or Gradient light interference microscopy (GLIM)^32^. Thus, our differential method, due to its low coherence (or long coherence length), gives us an advantage in rejecting out-of-focus light, suppressing speckles, and improving optical sectioning when we merge the spectral channels with the transport of intensity-based QPI.

To estimate the spatial frequency response of our QPI, we calculate the contrast transfer function as shown in Fig. 3e (bottom), estimating the contrast of the recovered phase ($\frac{{\phi }_{\max }-{\phi }_{\min }}{{\phi }_{\max }+{\phi }_{\min }}$) as a function of the spatial frequency (or radial position) in the Siemens star. For the ground truth, the contrast is fairly constant over all radii or spatial frequencies. For the metalens based QPI, the minimum lateral resolution is estimated to be $\sim 1\,{{\upmu m}}$ where the contrast curve dips under unity, comparable to the theoretical limit of $\frac{\lambda }{2{{\rm {NA}}}}=1.1\,{{\upmu m}}$.

The phase resolution, on the other hand, corresponds to the minimum axial or vertical topographical feature of the target, which can be resolved with QPI. For our measurement, targets less than 100 nm tall showed poor signal contrast (see S9), primarily limited by the low coherence of the LED source^46^ and noise—for e.g., thermal noise, dark current, photon shot noise, ambient noise in the room, etc. There is additional low-frequency numerical noise introduced by the Fourier domain solver for the TIE, as seen in the low-frequency regime of the contrast transfer function in Fig. 3e, or by large-scale non-uniformities of the recovered phase across the lateral dimension. We use a Tikhonov regularizer to guess the low frequencies in the recovered phase, which are lost due to the differential nature of the measurement. The value of the regularizer is kept constant through measurements. Note that the contrast curve for low spatial frequencies is often larger than unity due to this regularization in the TIE inverse solver, which prevents division by zero in the Fourier domain by adding a non-zero constant to the denominator, hence also artificially enhancing low-frequency components of the recovered phase (see “Methods” section—solving the TIE).

Even though the state-of-the-art interferometric methods may outperform the noise and phase sensitivity of our chromatic aberration-based QPI, we provide an advantage in terms of single-shot video-rate acquisition, tolerance to low coherence and fabrication imperfections, as well as phase imaging through coherent fiber bundles (CFB) which deterministically distort phase information. Yet, the achieved resolution of 0.1${\rm{\lambda }}$ height in vacuum (or $0.2{\rm{\pi }}$ phase) is sufficient for many in-vivo applications. Biological tissue in vivo will typically have refractive indices within 10% of water (${n}_{{{\rm {water}}}}$ = 1.33), hence falling within the range $n\in [\mathrm{1.33,1.47}]$^47^ with a maximum in-vivo refractive index contrast of $\delta n$ ~ 0.14 as opposed to $\delta n=0.5$ in our ex-vivo measurements. Hence, for the phase resolution limit in-vivo of $\phi =0.2\pi$, the optical path length sensitivity would correspond to ~ $\delta h=\frac{0.2\pi }{2\pi }(\frac{\lambda }{0.14})$ = 0.714${\rm{\lambda }}$ at best (about 380 nm for ${\rm{\lambda }}=530\,{\rm{nm}}$). Volumetric scattering, vibrations from biological rhythms, and ambient noise will further degrade in-vivo sensitivity to cell morphology-based phase modulations during real-time QPI. Nevertheless, given the QPI and optical sectioning capabilities of our method, we hope to achieve significant improvements in endoscopic contrast when imaging optically homogenous or non-absorbing tissue in vivo. Most human cells are 5–50 µm large^48^, with cell organelles being a few hundred nanometers in dimension (e.g. Ribosomes, the smallest organelles are ~50–100 nm in size). Hence sub-µm scale in-vivo quantitative phase imaging of single cells with methods that can detect phase through coherence destroying fiber bundles enables many diagnostic advantages - including observation of subcellular details in clinical investigation of clear tissue (such as lung or gut epithelia), imaging cellular scale dynamics in real-time, and better-targeted therapeutics in clear tissue.

As a final note, since propagation performs high-frequency filtering of the phase, smaller features can still be extracted using a larger number of defocus measurements^49^ along with higher order TIE that estimates the gradient using multiple defocus images (*n* > 3). For our spectral multiplexing approach, extra defocus measurements can be encoded into spectral channels using additional color filters at the detector or by employing a spectrally tunable source where the spectral lines correspond to different defocus distances.

##### Holographic reconstruction

Once the phase retrieval gives us the complex-valued electric field, we can reconstruct the entire 3D light field for a coherent beam. We can thus digitally refocus the object in the intermediate planes to extrapolate the scattered light field in the entire volume. Figure 3g shows reconstructed patterns for wavelengths in between the RGB measurements. Digital refocusing is computed as the 2D convolution of our phase object’s scalar, complex-valued electric field $\tilde{{E}_{o}}$ with the defocus PSF as $\tilde{E_{z}}\left(x,y\right)=\tilde{E_{o}}(x,y)* {h}_{z}(x,y)$, where ${h}_{z}(x,y)=\frac{{{\rm {e}}}^{{ikz}}}{i\lambda z}{{\rm {e}}}^{i\frac{2\pi }{\lambda }\frac{{x}^{2}+{y}^{2}}{2z}}$ is the Green’s function or PSF for defocus (assuming the propagation length is large, i.e., ${z}^{2} > > {x}^{2}+{y}^{2}$)^50^. The spectral-focal equivalence allows validation of the phase computed from the spectral channels by digital/numerical refocusing for various $\lambda$, followed by comparison with the measured defocus image stack for various $z$ (Fig. 3f). The through-focus image stack is considered as the ground truth for validation: we then computationally simulate equivalent spectral shifts and validate them against equivalent defocus planes by finding identical features. Hence, we are able to compute a hyper-spectral dataset starting from the single RGB image captured by the color camera, that can test the validity of our phase retrieval. Since phase retrieval is an ill-posed problem with multiple solutions satisfying given measurements, a holographic reconstruction, as proposed here, gives us a quantitative estimate of the accuracy of our solved light-field.

#### Quantitative phase endoscopy

The metalens microscopy setup is subsequently translated to a CFB endoscope by placing the metalens at the distal end of the endoscope. The metalens creates an image of the sample directly onto the planar distal facet of the CFB, which subsequently relays the image to the proximal end. The fiber bundle consists of 18,000 single fibers in a 1 mm^2^ area with a diameter of 3 µm each. The individual fibers within the bundle act as single pixels, retaining only the intensity of the incident light for each color while losing the information about direction ($\vec{\nabla }\phi$) or phase ($\phi$). However, in our scheme since the R, G, B intensity $I({\lambda }_{{\rm {r}}},{\lambda }_{{\rm {g}}},{\lambda }_{{\rm {b}}})$ channels encode phase $\phi$, we can afford to lose the coherent phase as long as the spectral channels are reliably transmitted through the fiber bundle, with the phase encoded in the RGB intensities. Additionally, fiber spectral intensity transmission is more resilient to bending and vibration. Hence, the spectral encoding of phase is more robust to real-time maneuvering than typical calibration-based methods that invert a transmission matrix which has to be re-calibrated every time the fiber moves or bends, or speckle-based methods that need large datasets^33^.

**Fig. 4:**
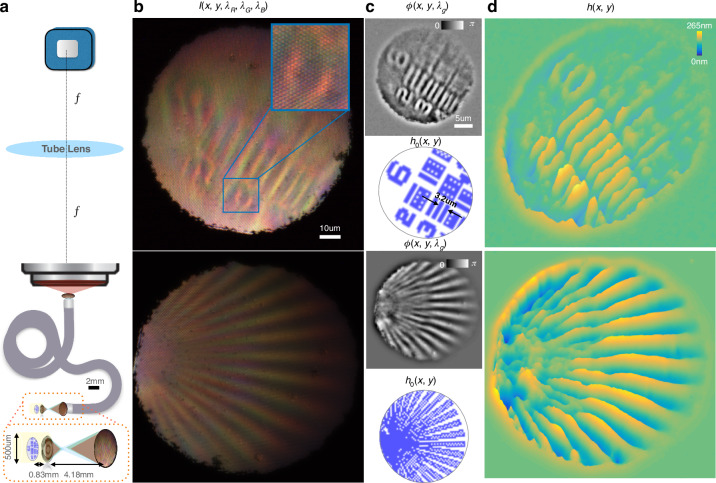
QPI through CFB. Validation of quantitative phase imaging through a coherent fiber bundle. The 3 µm individual fiber cores preserve spectral intensity but distort spatial phase. **a** The metalens magnifies the 250 nm tall calibration target by 5× onto the face of a coherent fiber bundle. The fiber bundle acts as a photodetector array at the distal end (facing the sample) and as an array emitter at the proximal end (facing the objective/camera). The image transmitted by the fiber bundle is relayed by a microscope objective to the color camera, with a further magnification of ×20. **b** The distal end of the fiber bundle preserves the intensity information for each color but scrambles the phase. Using the correlation between the color channels we can retrieve the quantitative phase of the sample as it was before being scrambled through the fiber. **c** Recovered quantitative phase at the distal end of the fiber along with the ground truth as measured by manufacturer Benchmark Technologies Inc. for two targets. Note that the target in the top row has a diagonal tilt that blurs the phase in the top right corner. **d** The height map is extracted as ${\phi }/\Delta {n}/{{k}}_{0}$ (${\delta }{n}=0.52,{{k}}_{0}=2{\pi }/530\,{\rm{nm}}$) at the nominal green wavelength. Scale bars are at the sample plane before magnification by the metalens

**Fig. 5:**
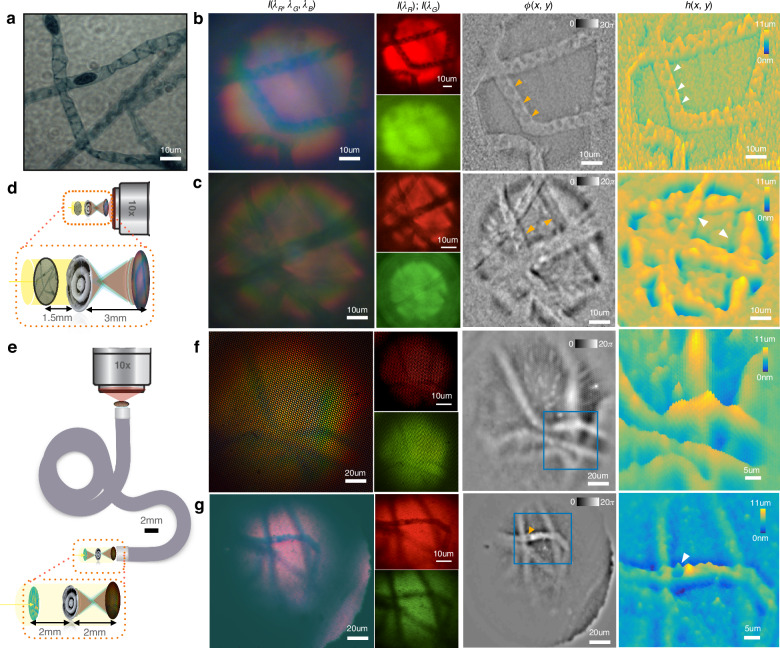
QPI for a Spirogyra sample. **a** Ground truth of the spirogyra algae shows spiral chloroplasts and dark beadlike zygoplasts. **b** and **c** From left to right—captured white light image ${I}({{\lambda }}_{{\rm{R}}},{{\lambda }}_{{\rm{G}}},{{\lambda }}_{{\rm{B}}})$, two color channels ${I}({{\lambda }}_{{\rm{R}}}){;I}({{\lambda }}_{{\rm{G}}})$, recovered quantitative phase ${\rm{\phi }}({x},{y})$, and height map ${h}({x},{y})$ respectively for metalens only imaging configuration **f**, **g** Corresponding images through the CFB endoscope. The recovered phase corresponds linearly to the optical density (${\delta }{n}\times {h}$) of the sample. **b** Metalens-based single-shot QPI shows structural information, such as individual chloroplast strands. **c** Spirogyra on either side of the focal plane show opposite phase curvature, seen as depressed or elevated grooves. **d** Imaging configuration for metalens only imaging system for **b**, **c** with the metalens magnifying the object by ×2 onto the fiber bundle. **e** Imaging configuration with metalens and coherent fiber endoscope for **f**, **g** this time with ×1 magnification. **f** QPI through an endoscope is able to distinguish top and bottom spirogyra in a crossing. **g** Structure of individual zygoplasts is visible in the phase image even in low-brightness conditions

With only a single color image, the phase is computed (via TIE) and validated with the ground truth (Fig. 4). For the first proof of principle, we assume transmission geometry and an extended point source. In an in-vivo setting, the transmission geometry can be achieved with oblique illumination, or by leveraging deeper reflective layers behind the tissue of interest. The lateral resolution is determined by the individual fiber core diameter (3 µm) or the numerical aperture of the metalens ($\frac{\lambda }{2{\rm {{NA}}}} \sim 1\,{{\upmu m}}$), whichever is larger. We choose an imaging geometry with ×5 magnification by the metalens onto the CFB with a calibration target (Fig. 4), where the magnification by the metalens is defined as the ratio of the image distance and the object distance (from the metalens surface). For our biological sample in Fig. 5, the magnification induced by the metalens onto the objective/fiber bundle is reduced to ×1/×2, respectively, for an increased field of view.

We finally image a volumetric biological target to extract structural and topological information from a single white light image. A spirogyra alga (*Spirogyra porticalis*) is chosen because of its distinct spiral chloroplasts, 10 µm, width resolvable by the endoscope, and three-dimensional winding geometry. Figure 5 shows recovered phase and height maps for the spirogyra, showing structural features such as strands in different planes and the topology of the spiral chloroplasts. With the endoscope added, although we lose resolution, we are still able to identify the elliptical nuclei (zygospores) and distinguish the top and bottom strands at a crossing. Coherent fiber bundles typically suffer from honeycomb artifacts due to the arrangement of the fibers in the bundle. Our single-shot phase retrieval automatically filters out the shared artifacts and common noise in the color channels due to the differential nature of its computation. The regularization in the solver effectively filters out fiber packing geometry in the recovered phase, while retaining the quantitative nature of the QPI due to calibration with a known target previously. The regularization parameter is kept fixed between the calibration and validation datasets, assuming all other experimental conditions remain the same. As also mentioned before, note that the regularizer adds numerical noise mostly in the low-frequency regime since the low frequencies are lost in the differential measurement. Additional frequencies can be recovered in a hyper-spectral dataset using multiple color channels at the camera or a swept source.

The phase resolution is within the lower and upper bounds determined by the noise and dynamic range of the camera respectively. Phase recovery with TIE has a distinct advantage in terms of imaging large structures in-vivo since the phase is automatically unwrapped when integrating the phase derivative in the TIE solver—as seen in the ~10$\pi$ phase range while imaging the spirogyra. For large phase gradients, the paraxial approximation, which assumes low angles of scattering, is weakened, potentially causing errors in the quantitative estimation of phase. Nevertheless, the paraxial approximation is well supported for our experimental paradigm, especially due to the low coherence of our white LED (which strengthens the paraxial approximation) and 0.2–20$\pi$ phase ranges in our samples. We found that our propagation-based QPI has a high dynamic range in the quantitative estimation of large phase variations while being sensitive to sub-micrometer phase fluctuations, along with the robust rejection of out-of-focus blur.

## Discussion

CFBs used in current endoscopy platforms can only image absorption in translucent tissue while treating phase effects such as refraction, diffraction, as noise, since optical coherence (and hence phase shifts) are deterministically distorted though the fiber bundle. Please note that the “coherent” in CFB refers to the physical coherence of coplanar single-mode fiber facets constituting the bundle, whereas the optical coherence is distorted by nanometer-scale inhomogeneities between adjacent fibers. Integration of our proposed QPI with existing endoscopy or ophthalmologic imaging architectures would enable the extraction of optical density maps of clear layers of tissue while rejecting out-of-focus blur. Towards this goal, we have reported a single shot technique for recovering the quantitative phase for suture-less endoscopy (<1 mm incision) with a single metalens at the distal tip of the endoscope. We utilize the chromatic dependence of focal distance inherent in a hyperboloid metalens to encode three defocus planes into RGB color channels before transmission through an endoscope. The color channels are measured simultaneously by a color camera after transmission; the through-focus intensity enables computational recovery of the quantitative phase. The inherent defocus chromatic aberrations in hyperboloid metalens are fortuitously tailored to have an exact inverse linear dependence for the three imaging wavelengths with the focal distance. Our method is shown to be applicable with off-the-shelf white light LEDs and any off-the-shelf color camera, enabling potential translation to mobile Point of Care Diagnostics^51^ platforms, or to existing commercial endoscopes, with a single additional optical element and minimal computational overhead. Our phase resolution and field-of-view depend upon the numerical aperture of our metalens and the quality of fabrication of the nanostructures, sufficient in our prototypes to resolve subcellular detail in ex-vivo trials. Since only a single metalens is used, our design can be easily translated to commercial holographic endoscopy, which captures the entire 3D light field, even for low coherence or high dispersion conditions. The phase contrast mechanism is sensitive to a tenth of wavelength in the presence of noise at room temperature, with nominal (<100 ms) camera exposure time to support >10 Hz video rate acquisition for a 27 mW white light LED. Our single shot QPI’s phase resolution is limited by the noise, coherence, and fabrication tolerances to about $0.2\pi$. The lateral resolution was seen to be diffraction-limited by the metalens aperture at $\lambda /2\,{\rm {{NA}}}=1\,{{\upmu m}}$ for the microscopy configuration and by the fiber core diameter (3 µm) in the endoscopic configuration with a fiber bundle. Hence, our QPI-enabling hyperboloid metalens can be added onto any endoscopic modality, maintaining the native resolution of the coherent fiber bundle, as long as the metalens has a higher resolution than the individual fibers in the CFB.

Our emphasis here is on easy translational potential to clinical endoscopy, even with low coherence, high ambient noise, or poor manufacturability. Due to the simplicity of the hyperboloid lens design, widely available CMOS color sensors, as well as the fast Fourier domain processing algorithm, our QPI strategy is easily translatable to clinical or in-vivo applications where the tissue being probed is too transparent for conventional methods. Several challenges still need to be addressed for translational applications of our method. The single lens system used for phase imaging is simple in terms of alignment and complexity, yet suffers from non-tele-centricity that has to be compensated computationally^52^. In a single lens system, the image is formed on a spherical wavefront—the magnification varies as a function of the field of view (lateral) as well as defocus (transverse). Hence the magnification varies slightly between the red, green, and blue channels, causing a spherical aberration in the phase. A potential solution would use a grin lens at the distal end of the endoscope to collimate the wavefront from the metalens and ensure tele-centricity.

Our proof of concept, like most QPI imaging architectures, is implemented in single-pass transmission geometry. In-vivo applications, however, predominantly require reflection mode imaging. Quantitative phase information gets blurred in double-pass reflection geometry due to multiple scattering within in-vivo samples. There are two ways to use deeper reflective layers for in-vivo QPI. On the one hand, interference effects for phase collected during the inward propagation of light in a tissue can be captured on an effective screen in the presence of opaque membranes or diffuse layers embedded in the tissue being imaged, like in the mammalian retina^27^. Alternately, by localizing or focusing an excitation probe deep inside a fluorescing or auto-fluorescing layer of the tissue^30,31^ or by using oblique illumination angles^28,29^, single-pass transmission illumination can be achieved using the return path of the light out of the tissue. In either case, as long as there is sufficient back reflection from a tissue underlying the structure of interest, we will be able to extract a meaningful QPI signal using our strategy described here.

Various future directions will help accelerate the translation of our metalens-based QPI to relevant applications. For confocal point scanning systems, our metalens can be directly added at the pupil or scanning mirror in the illumination path to recover the phase from spectral channels. For conventional flood illumination, the metalens has to be in the collection path, with the tissue of interest sandwiched between the back reflecting deeper layers and the metalens detection plane. Additionally, by including more spectral control or tunability at the source or detector, using either a supercontinuum laser or a spectrometric detector, we can potentially capture multiple defocus planes with depth resolution limited only by the spectral linewidth—opening the doors for spectral coherence-based full tomographic QPI, similar to higher order TIE with multiple defocus planes^49^.

## Materials and methods

### Solving the transport of intensity equation

For our chromatically aberrated hyperboloid metalens, the chromatic wavelength is exactly linear with the inverse focal length, hence $\lambda z={{\rm {constant}}}$. Taking the derivative shows that *λ* d*z* = *−z* d*λ*. To estimate the left-hand side of the TIE equation, we can either estimate $\frac{{{\rm {d}}I}}{{z{\rm {d}}}\lambda }$ or $\frac{{{\rm {d}}I}}{\lambda {{\rm {d}}z}}$, as both are equivalent. The numerator ${{\rm {d}}I}$ is the difference between the images for either two color channels or two defocus channels. For the first case, ${\rm {d}}\lambda$ is the difference in the corresponding wavelengths at nominal focal distance *z* = 1.15 mm. Identically, for the second case similar to conventional defocus based TIE, we can use ${{\rm {d}}z}=-\frac{{z{\rm {d}}}\lambda }{\lambda }$ at nominal wavelength of $\lambda =530\,{\rm {{nm}}}$. Next, to solve the TIE, we substitute the Poynting vector by the gradient of an arbitrary scalar potential $\psi$ as $I\vec{\nabla }\phi =\vec{\nabla }\psi$ (Helmholtz decomposition), to arrive at a Poisson equation of the form ${{\rm {d}}I}/{\rm {d}}\lambda ={\nabla }^{2}\psi$. First, the Laplacian ${\nabla }^{2}$ is integrated into the Fourier domain. The double derivative in the two-dimensional Laplacian ${\nabla }^{2}={\nabla }_{x}^{2}+{\nabla }_{y}^{2}$ Fast Fourier transforms (FFT) into a parabola (${k}_{x}^{2}+{k}_{y}^{2}$). By dividing the gradient by ${k}_{x}^{2}+{k}_{y}^{2}$, with a constant regularizer $\epsilon$ added to prevent division by zero at the bottom of the parabola, we can calculate $\psi$ as $\frac{FFT({{\rm {d}}I}/{{\rm {d}}z})}{{k}_{x}^{2}+{k}_{y}^{2}+{\rm{\epsilon }}}$. Subsequently, $\phi$ is similarly solved from $\psi$ by inverting another Laplacian ${\nabla }^{2}\phi =\vec{\nabla }.\left(\frac{\vec{\nabla }\psi }{I}\right)$, using a second regularizer $\epsilon {\prime}$. The regularization parameters $\epsilon ,\epsilon {\prime}$ are held constant between the calibration step with the known target and measurements with the sample. An iterative procedure then updates a residue in the intensity gradient by forward propagating the solved fields via the TIE and TPE. The residue is solved again with the TIE solver to give a corrected phase estimate, which is applied repeatedly until convergence. The iterative solution helps remove artifacts in the presence of absorption, recovers vorticity, and improves the fidelity of the quantitative phase^44^. Importantly, the phase recovered by inverting the TIE is unwrapped by the integration steps, removing the constraint $\phi \in [\mathrm{0,2}\pi ]$. Hence large values of the refractive index or height variations are encoded directly into the recovered phase as large phase values without the typical phase wrapping seen in interferometric approaches to QPI, which reduces the overall computational cost. Finally, each iteration of the solver needs at most four Fourier transforms whose computational complexity scales as O(*n* log_2_ *n*) with the image size (*n*), allowing real-time computational even on mobile computing platforms or in a point-of-care setting with limited computation.

### Metalens fabrication

We fabricated the metalens using a 500-micron-thick fused silica wafer and deposited 600 nm of Silicon Nitride using Plasma-enhanced chemical vapor deposition at 350 °C. A 300 nm-thick layer of ZEP 520A followed by a thin film of anti-charging polymer (DisCharge H_2_O) was spun coat on top of Si_3_N_4_ thin film. Next, the lens patterns are written by electron beam lithography (JEOL 6300) at a beam energy of 100 keV, beam current of 8000 pA, and a base dose of 275 µC/cm^2^ and appropriate proximity effect corrections. The resulting designs are developed in a solution of amyl acetate and Iso-Propyl alcohol. The exposed and developed samples are then placed in a physical evaporator to deposit roughly 50 nm of aluminum oxide by electron beam evaporation. The dissolution of the remaining resist performs lift-off in N-Methyl-2-pyrrolidone (NMP) at 90 °C for 12 h. Finally, the pattern is transferred from the aluminum oxide mask to the Si_3_N_4_ by using a fluorine-based RIE process (Oxford) leaving a total thickness of 10 nm of Alumina over 600 nm of Si_3_N_4_.

### Metalens characterization

We characterized the metalens with RGB fiber-coupled LEDs from Thorlabs. Red M625F2, Green M530F2, and Blue M455F3 LEDs are coupled to a 100 µm single fiber core. The collimating lens is chosen to be $2^{\prime\prime}$ diameter, 30 mm focal length achromat. For enhanced signal-to-noise ratio in the phase retrieval, higher coherence is preferred at the cost of more speckles in the image. Increasing coherence with a smaller diameter fiber and longer focal length collimating lens at the source would yield sharper phase images since lower coherence effectively leads to image blurring. However, higher coherence leads to lower photon count, hence requiring larger camera integration time. The imaging is performed with a ×20 objective and a standard 150 mm tube lens. The model of the color camera used is Allied Vision GT1350.

### Imaging setup

Imaging experiments are performed with collimated Light emitted from the Thorlabs white light fiber coupled LED (MCWHF2) incident on the sample, followed by imaging by the meta-lens. The LED is operated at nominal power (~27 mW) spread over a 1 inch collimated beam in flood illumination for a power concentration of ~55 W m^−2^, well below the acceptable range for most tissue in the body, including the eye, for a limited duration.

Since we are utilizing chromatic aberrations, a particularly useful alignment strategy with white light is by making sure that the focal spot for one color is co-centric with the other two colors when the sample is removed from the system, and the microscope is imaging the metalens focal plane. In the case of misalignments, the chromatic focal spots will shift laterally and lose co-centricity. The spectral-focal calibration is performed with a Newport 10DKIT-C1 ${5}^{\circ }$ precision diffuser, characterized by a ${5}^{\circ }$ beam waist of the scattered beam for a plane wave incident on the diffuser. The phase calibration sample is manufactured by Benchmark Technologies Inc. The image plane is relayed by the ×20 objective to the camera. The ground truth phase measurement for the calibration target is performed with the Phasics^©^ SID4 HR phase imaging camera attached at the output port of a regular transmission microscope.

The metalens imaging setup includes an infinity-corrected ×20 objective (Nikon Plan Fluor ×20, 0.50 NA), which collects light scattered by the metalens at a distance of 2 mm from the metalens. The objective and tube lens assembly enables a ×20 magnification of the image formed by the metalens. The tube lens is from Thorlabs (AC254-200-A-ML) with focal length *f* = 200 mm. The sensor is 3.2 MP Infinity5-3 CMOS camera with an effective RGB pixel size of 3.2 µm. With a magnification by a ×20 objective between the image formed by the metalens and the camera’s CCD array, the effective pixel size at the image plane is 0.16 μm. Since our images have an NA limited resolution of >1 µm, the image is well resolved by the color camera + objective combination. For ×5 or ×40 objectives, the pixel size at the image plane is 0.64 or 0.08 µm, respectively, sufficient for resolving the image formed by the metalens. Since the resolution limiting step in the system is at the metalens and not the objective, the only consideration while switching objectives is to ensure that the field of view includes the entire fiber tip (especially for higher magnifications >×40).

Frame rates for all the measurements are >10 Hz, with all three color channels captured in a single acquisition, enabling real-time phase imaging in an in-vivo setting. Due to the relatively simple algorithm that involves only two fast Fourier transforms to solve for phase, we have minimal computational lag in the QPI algorithm.

For imaging with an optical fiber (Schott RLIB CVET, 1.05 × 910, 7.6M, 18K19, QA.90) with the distal end facing the metalens and the proximal end relayed by the microscope to the camera, both ends of the fiber are placed on a 3D kinematic stage for alignment; the sample and the metalens are also on kinematic stages. An alternative strategy with fewer moving parts uses a 2 mm-thick O-ring to separate the metalens and the sample, which are then mounted with scotch tape in a fixed imaging configuration (see Supplement S2 for full schematic). Spirogyra samples were purchased from Carolina Biological Supply (# 296548). Two slides are stacked face to face with the Spirogyra sandwiched between the glass to accentuate 3D structures. The distal end of the fiber looks at the sample through the metalens; the proximal end of the coherent fiber bundle is directly observed under a Nikon Eclipse LV100 microscope with the objective-dependent magnification as indicated in the figures.

### Estimating effective wavelengths for phase retrieval

The quantitative phase retrieval needs accurate values of the effective wavelengths ${\lambda }_{{\rm {r}}},{\lambda }_{{\rm {g}}},{\lambda }_{{\rm {b}}}$ for the RGB intensity channels of the color camera, respectively. Since we use a broadband white light LED source, the camera and source spectral curves have to be multiplied to find the peak effective wavelengths corresponding to Red, Green, and Blue. We use a spectrometer to measure the source emission spectrum, which is multiplied by the standard response curve of the camera’s Bayer color filter to find our peak wavelengths to be almost identical to the wavelengths used for metalens characterization i.e. 625, 530, and 455 nm, respectively. Since the emission is from an LED source, the source spectral channels are fairly broadband. Hence the effective spectral linewidth of each color channel is primarily determined by the camera’s spectral filters. Transport of intensity-based QPI is extremely robust to partial coherence and broad linewidths as long as the spectral channels are centered at the assumed values in the inverse solver and the regularizer has been calibrated with a known target. Additionally, the long coherence length due to the broadband sources also helps with reducing speckle and noise.

## Supplementary information

Supplemental Information for Quantitative Phase Imaging Endoscopy with a Metalens
