# Supplementary material for: Quantitative phase imaging endoscopy with a metalens
Source: Light Sci Appl. 2024 Nov 8;13:305. doi: 10.1038/s41377-024-01587-y (PMC11543855; doi:10.1038/s41377-024-01587-y)
Supplement: Supplementary file 1 — Supplemental Information for Quantitative Phase Imaging Endoscopy with a Metalens [file 41377_2024_1587_MOESM1_ESM.pdf]

Supplementary Information for Quantitative Phase Imaging Endoscopy with a Metalens

Aamod Shanker,[1,4], Johannes Fröch[1], Saswata Mukherjee[1], Maksym Zhelyeznyakov[1], Steven Brunton[3], Eric Seibel[3], Arka Majumdar[1,2]

[1]Department of Electrical and Computer Engineering, University of Washington, Seattle, WA, 98195, USA

[2]Department of Physics, University of Washington, Seattle, WA-98195 USA

[3]Department of Mechanical Engineering, University of Washington, Seattle, WA 98105, USA

[4]Center for Vision Science, University of Rochester, New York, NY 14623, USA

* Corresponding author: arka@uw.edu

S1: Derivation of the Transport of Intensity and Phase from Maxwell's equations.

We now derive the transport equations for free space optical propagation under the paraxial approximation with some approximations to the fundamental equations of Maxwell. Electromagnetic waves propagating in free space were first described by the ubiquitous coupled differential equations Maxwell's equations. The corresponding Helmholtz Equation is derived for a harmonic wave from Maxwell's equation:

$$({\nabla_{xyz}}^2 + k^2)\hat{E} = 0 \quad \text{——} (1)$$

where $\hat{E}$ is shorthand for $\hat{E}(x, y, z)$, the electric field vector distribution in space, k is the spatial momentum or wave-vector with x, y, z components given by k_x, k_y, k_z respectively, and $|k| = 2\pi/\lambda$. Additionally, $k^2 = k_x^2 + k_y^2 + k_z^2$ is the conservation law for the propagation vector and the operator ${\nabla_{xyz}}^2 = \nabla_x^2 + \nabla_y^2 + \nabla_z^2$ is the 3D scalar Laplacian.

Also $\hat{\nabla} = \frac{\partial}{\partial x}\hat{x} + \frac{\partial}{\partial y}\hat{y}$ is the 2D in-plane gradient vector and $\nabla^2 = {\nabla_{xy}}^2 = \nabla_x^2 + \nabla_y^2$ is the 2D scalar Laplacian. Under the paraxial approximation where the light is principally propagating along z, $k_z >> k_X^2 + k_y^2$. Hence the electric field can be approximated as $\vec{E}(x, y, z) = U_0(x, y, z)e^{ikz}(\hat{x} + \hat{y})$, with the electric field polarized normal to propagation in z.

Substituting into the equation (1) and expanding the z derivative by the chain rule,

$$(\nabla_{xy}^2 + \nabla_z^2 + k^2)U_0(x, y, z)e^{ikz} = 0$$

$$\Rightarrow [\nabla_{xy}^2 U_0 - k^2 U_0 + 2ik\frac{dU_0}{dz} + ik\frac{d^2U_0}{dz^2} + k^2 U_0]e^{ikz} = 0$$

$$\Rightarrow [\nabla_{xy}^2 U_0 + 2ik\frac{dU_0}{dz} + ik\frac{d^2U_0}{dz^2}] = 0$$

Further assuming that due to the paraxial approximation, the field is slowly varying in the propagation direction, i.e. $\frac{d^2U_0}{dz^2} \to 0$

$\Rightarrow \nabla_{xy}^2 U_0 + 2ik\frac{dU_0}{dz} = 0$ —— (2)

which is called the paraxial form of the Helmholtz equation and describes the evolution of the wave along the propagation direction z.

Since $U_0(x, y, z)$ is a complex valued scalar field, it can be represented as its amplitude and phase components, $U_0(x, y, z) = A(x, y, z)e^{i\phi(x,y,z)}$ —- (3)

Substitute the complex field (3) into the paraxial Helmholtz equation (2), we obtain:

$$\nabla_{xy}^2 Ae^{i\phi} + 2ik\frac{dAe^{i\phi}}{dz} = 0$$

$\Rightarrow [\nabla^2 A - (\hat{\nabla}\phi)^2 A - 2k\frac{d\phi}{dz}A] + i[A\nabla^2\phi + 2k\frac{dA}{dz} + 2\hat{\nabla}A.\hat{\nabla}\phi] = 0$ —- (4)

Both the real and imaginary parts of Eqn. (4) must be zero.

$\Rightarrow [\nabla^2 A - (\hat{\nabla}\phi)^2 A - 2k\frac{d\phi}{dz}A] = 0$ —— (5) is the real part of (4)

$\Rightarrow i[A\nabla^2\phi + 2k\frac{dA}{dz} + 2\hat{\nabla}A.\hat{\nabla}\phi] = 0$ —— (6) is the imaginary part of (4)

The imaginary part of the Helmholtz equation or Eqn. (6), can be reduced to the *Transport of Intensity* equation by multiplying by $A(x, y, z)$ on both sides,

$$[A^2\nabla^2\phi + 2kA\frac{dA}{dz} + 2A\hat{\nabla}A.\hat{\nabla}\phi] = 0$$

$$\Rightarrow [A^2\nabla^2\phi + k\frac{dA^2}{dz} + \nabla A^2\hat{\nabla}\phi] = 0$$

Since the square of the amplitude is the photon intensity, i.e., $A^2(x,y,z) = I(x,y,z)$ we obtain,

Transport of Intensity Equation

$$\Rightarrow [I\hat{\nabla}^2\phi + k\frac{dI}{dz} + \hat{\nabla}I.\hat{\nabla}\phi] = 0$$

$$\Rightarrow \hat{\nabla}_{xy}.I\hat{\nabla}_{xy}\phi = -k\frac{dI}{dz} \qquad \text{——- (7a)}$$

Eqn. (7a) is the Transport of Intensity equation that describes the evolution of intensity longitudinally along *z* with the gradients of phase and intensity in the transverse (*x*-*y*) plane. Hence, it encapsulates such effects as refraction through a surface of arbitrary shape, as well as wavelength dependent color splitting with a prism, focusing by a lenslet array etc. However optical phenomena such as diffraction from an occluding edge are missed by the TIE, but captured by the Transport of Phase equation instead, as described below.

The Transport of intensity if analogous to the **Continuity Equation in fluid dynamics** – if ρ is the fluid density, v is the fluid velocity vector, and t is time, then by conservation of fluid mass,

$$\hat{\nabla}.(\rho\hat{v}) = \frac{d\rho}{dt} \qquad \text{——— (7b)}$$

If we substitute $I \rightarrow \rho$, $\hat{\nabla}\phi/k \rightarrow \hat{v}$ and $dz \rightarrow dt$, we arrive at the equivalence of (7a) and (7b). Hence the laws of fluids and of light intensity propagation are homologous, with optical intensity equivalent to fluid density, and phase gradients corresponding to fluid velocity.

Next, we describe the equivalence of transport of momentum ($\rho\hat{v}$) or phase gradients ($\hat{\nabla}\phi$). The transport of momentum equations in fluids is called the Euler equation. We multiply the real part of the Helmholtz equation Eqn. (5) by $A(x,y,z)$,

$$\Rightarrow [A\hat{\nabla}^2 A - (\hat{\nabla}\phi)^2 A^2 = 2k\frac{d\phi}{dz}A^2]$$

$$\Rightarrow [\frac{\nabla^2 A}{A} - (\hat{\nabla}\phi)^2 = 2k\frac{d\phi}{dz}]$$

substitute $A = \sqrt{I}$ and expand the derivatives in terms of I and ϕ to obtain the

Transport of Phase equation

$$\frac{\nabla^2 I}{I} - \frac{(\hat{\nabla}I)^2}{2I^2} - (\hat{\nabla}\phi)^2 = 2k\frac{d\phi}{dz} \text{ ———(8a)}$$

The Transport of Phase Equation (TPE) for light is analogous to the model for non-viscous fluids (Euler Equation) since they both describe direction of flow as velocity ($\vec{v}$) or momentum ($I\hat{\nabla}\phi/k$) evolution respectively.

To draw the comparison between fluid mechanics and electromagnetics, apply a spatial derivative $\hat{\nabla}(Eqn. 8a)$ to obtain:

$$\frac{\hat{\nabla}\nabla^2 I}{I} - 2\frac{\nabla^2 I}{I^2}\hat{\nabla}I - \frac{2\hat{\nabla}I\nabla^2 I}{2I^2} + \frac{(\hat{\nabla}I)^2}{I^3}\hat{\nabla}I - 2(\hat{\nabla}\phi.\hat{\nabla})(\hat{\nabla}\phi) = 2k\frac{d\hat{\nabla}\phi}{dz}$$

Dropping all third order derivatives by assuming slow changes in intensity; $\nabla^n I \to 0$ where $n > 2$

$$-\frac{\nabla^2 I}{I^2}\hat{\nabla}I + \frac{(\hat{\nabla}I)^2}{I^3}\hat{\nabla}I - (\hat{\nabla}\phi.\hat{\nabla})(\hat{\nabla}\phi) = k\frac{d\hat{\nabla}\phi}{dz} \text{ —— (8b)}$$

The Euler Equation is given by:

$$-\frac{\hat{\nabla}p}{\rho} - (\vec{v}.\hat{\nabla})\vec{v} = d\vec{v}/dt \text{ —— (8c)}$$

where p is the internal pressure, ρ is the fluid density and $\vec{v}$ is the velocity. Substituting $p \to \frac{1}{k^2}\frac{\hat{\nabla}I}{I}$, $\rho \to I$ and $\vec{v} \to \hat{\nabla}\phi/k$, the modified Euler equation (Eqn. **8c)** becomes

$$-\frac{\nabla^2 I}{I^2}\hat{\nabla} I + \frac{(\hat{\nabla} I)^2}{I^3}\hat{\nabla} I - (\hat{\nabla}\phi.\hat{\nabla})\hat{\nabla}\phi = k\frac{d\hat{\nabla}\phi}{dt} \quad \text{—— (8d)}$$

Comparing (8b) and (8d), we observe the exact isomorphism between the paraxial transport equation for optical phase and the Navier Stokes' equation for transport of momentum in fluids.

The equivalence between light and fluid transport equations is arrived at by substituting the pressure term $p \rightarrow \frac{\hat{\nabla} I}{I}$ i.e. the pressure exerted by photons is proportional to the derivative of intensity, well known in optical trapping literature.

Additionally, since we are measuring light at steady state conditions, the dynamic variable in the Transport of Phase (Eqn. **8a**) is defocus (*z*) instead of time (t). Also, $(\hat{\nabla}\phi)^2 = \hat{\nabla}\phi.\hat{\nabla}\phi$ is the divergence or Bernoulli term, that relates to the rate of outward expansion along velocity vectors. Hence light can be mathematically modeled as a non-viscous fluid that exerts pressure along intensity gradients ($p \rightarrow \frac{1}{k^2}\frac{\hat{\nabla} I}{I},$) and has flow along its phase gradients ($\vec{v} \rightarrow \hat{\nabla}\phi/k$).

S2: Full Measurement Setup

The measurement was performed directly on a Nikon microscope for the data collection, with an external LED illumination source built next to the microscope. The metalens to sample distance was adjusted in steps of 1 mm by using a single or multiple one-inch O-rings (thickness of 1 mm) as spacers. The fiber was mounted on the microscope stage using an LCM-2 self-centering lens mount that can hold the fiber tip securely. Figure S1 shows the measurement setup.

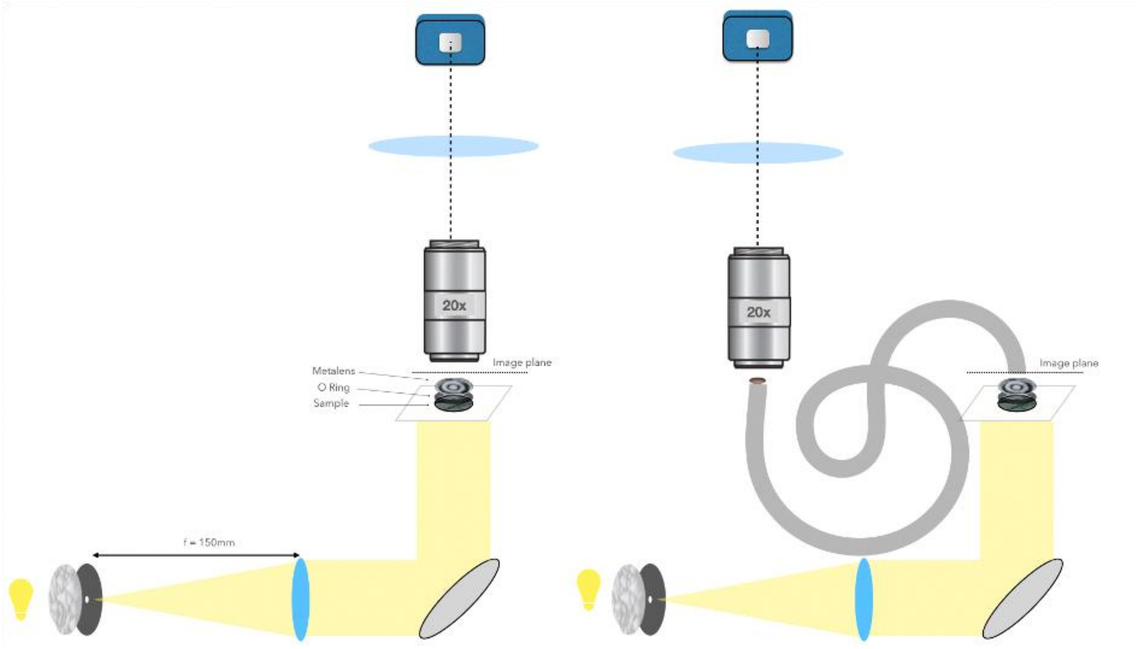

Figure S1 Full measurement setup

Metalens Microscopy (left): An incandescent lamp or LED is used as a broadband source, followed by a ground glass diffuser and a pinhole aperture to ensure a homogeneous beam. A collimating lens is placed at 150 mm from the pinhole, followed by a mirror that illuminates the sample from underneath. The metalens is placed two focal lengths from the sample at the image plane, forming an image at the working distance plane of the objective. The objective and 150 mm tube lens magnify the image formed by the metalens onto the camera by 20x (objective dependent). In certain configurations, an O-Ring is used to mount the metalens onto the sample with scotch tape at 1 mm or 2 mm. **Metalens Endoscopy (right):** The image formed by the metalens is relayed by the coherent fiber bundle to the microscope objective, which forms an image of the proximal tip of the fiber bundle at the camera. The resolution is limited by the size of each coherent fiber in the bundle, which is about 10 um.

S3 : Estimating aberrations in metalens imaging

Using a continuous transformation between the measured images of a precision diffuser with a microscope vs a metalens, we can get an intuitive estimate of the aberrations introduced by the metalens in our images. The comparison is performed digitally, by focusing the microscope objective first on the diffuser (first column in Figure S2) and then adjusting the working distance to the image formed by the metalens (fourth column in Figure S2). The intermediate images are produced by a digital superposition of two images. The ground truth image is gradually morphed into the corresponding metalens image as a linear superposition: $\alpha I_{metalens} + (1 - \alpha) I_{defocus}$; $\alpha \in (0,1)$. The top row corresponds to positive defocus (microscope) or green channel (metalens) and the bottom row corresponds to the negative defocus (microscope) / red channel (metalens). We utilize this empirical comparison to estimate the quality of our metalens fabrication and to potentially quantify the aberrations in the metalens based imaging system (or the Contrast Transfer Function) based on PSF deformations at the junctions of the caustic lines.

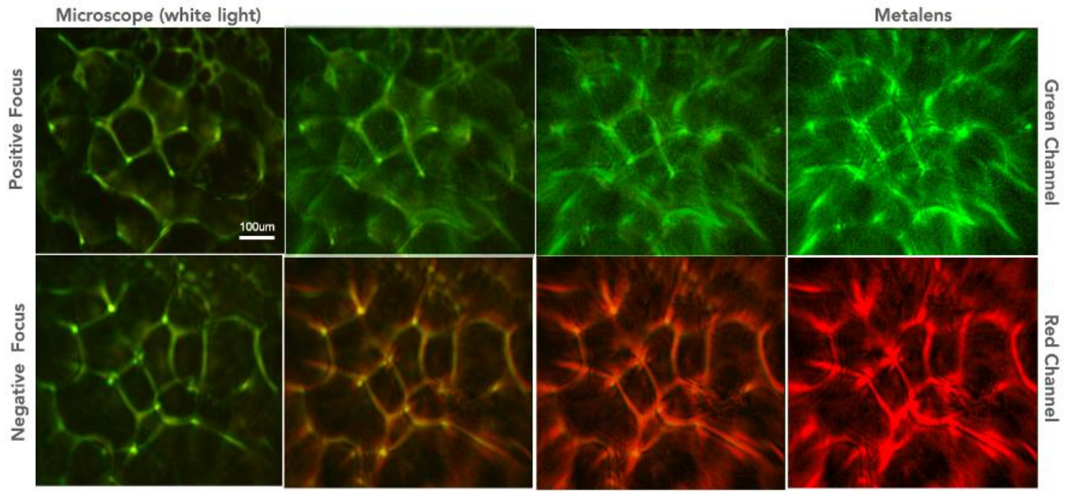

Figure S2. Comparison of caustics: Microscope defocus vs Metalens Color Channels. The top row is the positive defocus in the microscope (left) as it morphs into the green channel of the metalens (right). The bottom row corresponds to negative defocus in the microscope (left) morphing into the red color channel in the metalens image (right). The columns in between the left and right show a continuous transformation starting from the ground truth microscope images to the aberrated images formed by the metalens.

S4 : Rotation of the Seimen's star between metalens spectral channels

Since our metalens spectral channels encode equivalent defocused images, we can observe a through-spectrum rotation of the Seimen's star target used to calibrate the QPI. Like linear gratings that shift along the grating vector on propagation or diffraction, a circular spoked wheel turns about its center. Thus, angular momentum is imparted to the beam, which is seen in the raw white light image formed by the metalens as three slightly turned, overlapping spoked wheels for each color channel (Figure S3). We measure this rotation between the spectral channels, as shown in the Figure S3.

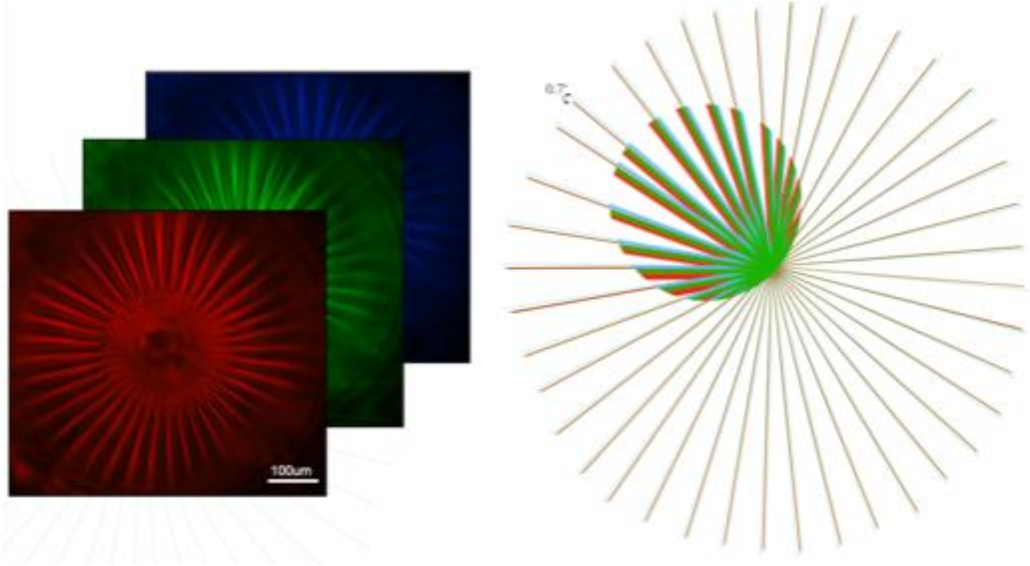

Figure S3. Spoke locations vs Spectrum for Seimen's Star target: The outermost spokes in Seimen's star are represented as colored lines to demonstrate the rotation of the Seimen's star target with RGB color / wavelength, identical to its diffraction behavior through-focus. A manual fitting shows an angular rotation of 0.7° between the green and blue channels. The center is magnified to show detail.

S5: Design of the meta-optic / SEM of fabricated metalens

The computational design of the metalens relies on Rigorous Coupled Wave Analysis (RCWA) simulations of the phase shift and transmission of periodic arrays of square nano-pillars. The simulations were performed under periodic boundary conditions assuming an infinite array of fixed sized pillars. The crosstalk between different sized pillars is assumed to be negligible, which is valid for a quasi-periodic distribution of scatterers with adjacent pillars having similar dimensions. With a fixed periodicity and pillar height, each meta-atom is simulated by sweeping the pillar width to search for the desired phase shift. After fabrication, most of the pillars are qualitatively more similar to cylinders than squares (Figure S4). The metalens focal distance is subsequently characterized with respect to the RGB wavelengths and shown in Table S1.

Table S1. Hyperboloid lens: measured focal length dispersion shows that for our hyperboloid metalens the product of focal length and wavelength λf is within 0.49% for three visible color channels.

λ(nm)	*f (mm)*	λf (nm-mm)
625	0.970	606
530	1.155	609
455	1.347	609

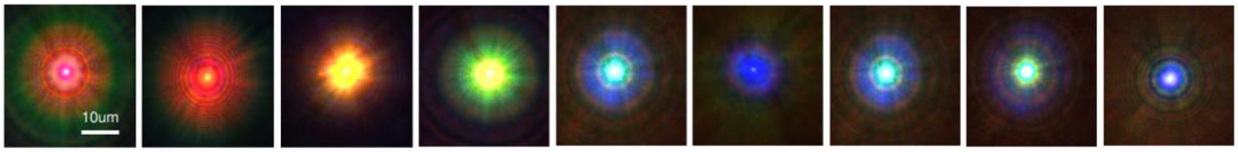

Focus spot vs depth ($\delta z = 50um$ steps) with white light

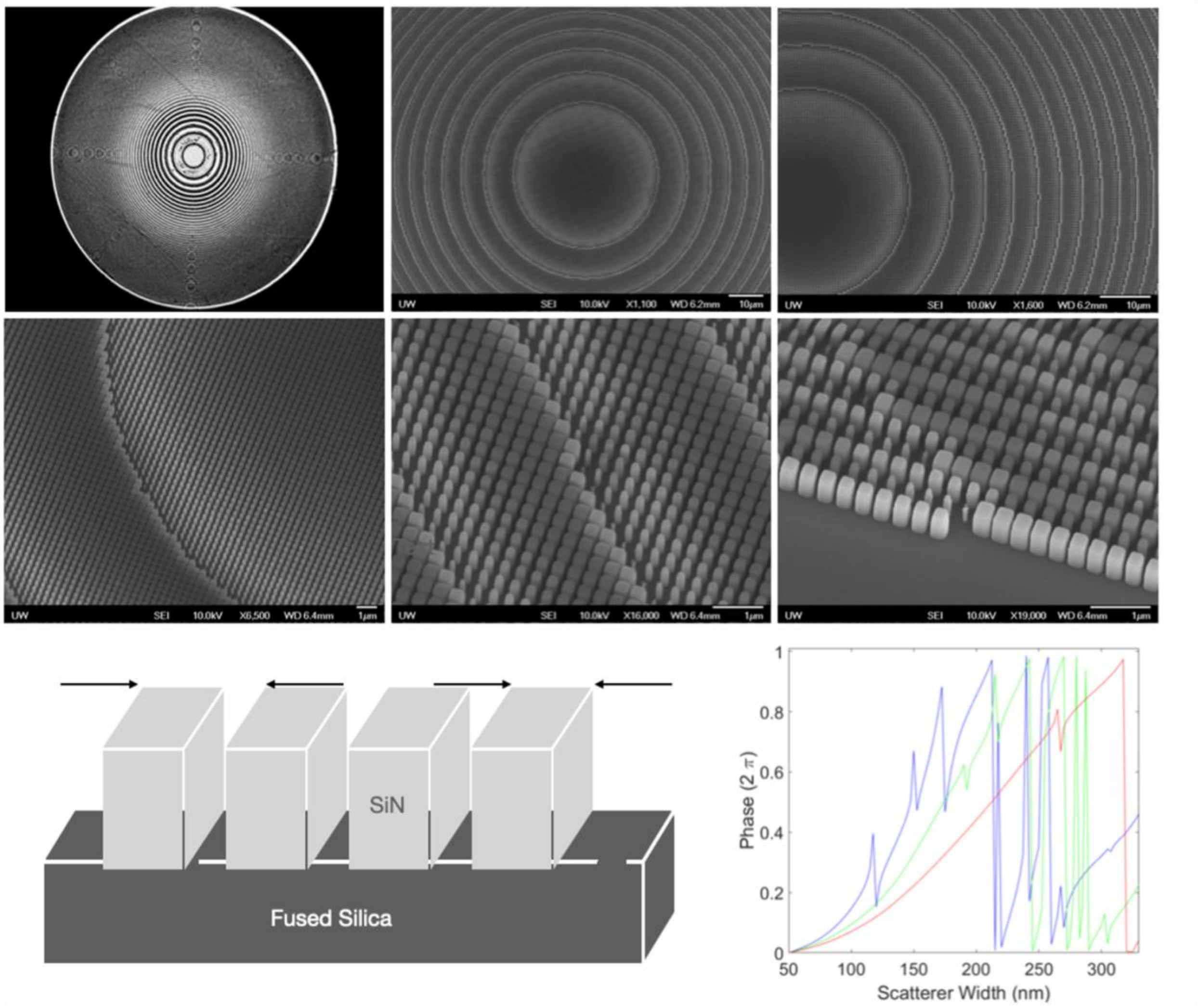

Figure S4. Top two rows: Scanning Electron Microscopy of fabricated metalens shows the nano-pillars at various resolutions; scale bars at bottom right. **Bottom row:** Metalens design estimates the phase shift for each color (blue, green, and red correspond to 455 nm, 530 nm and 625 nm wavelengths respectively). The simulation assumes square pillars and S polarized light (equivalent for P polarization under normal incidence due to the square geometry). Pillars are chosen to impart desired phase shift according to the hyperboloid phase profile at the green wavelength, taking care to avoid regions with strong fluctuation.

S6: Single Shot metalens QPI with a binary amplitude target through a fiber bundle

To show the feasibility of the chromatic aberration-based phase retrieval by using our metalens, our first experiment was rather simple – using a binary amplitude target (clear features with a black background) to test if color channels show any differences when imaging with a metalens. We were able to leverage chromatic focusing differences off the edge of the feature to recover the trivial phase of the US test target, paving the way for more intricate phase objects (Figure S5).

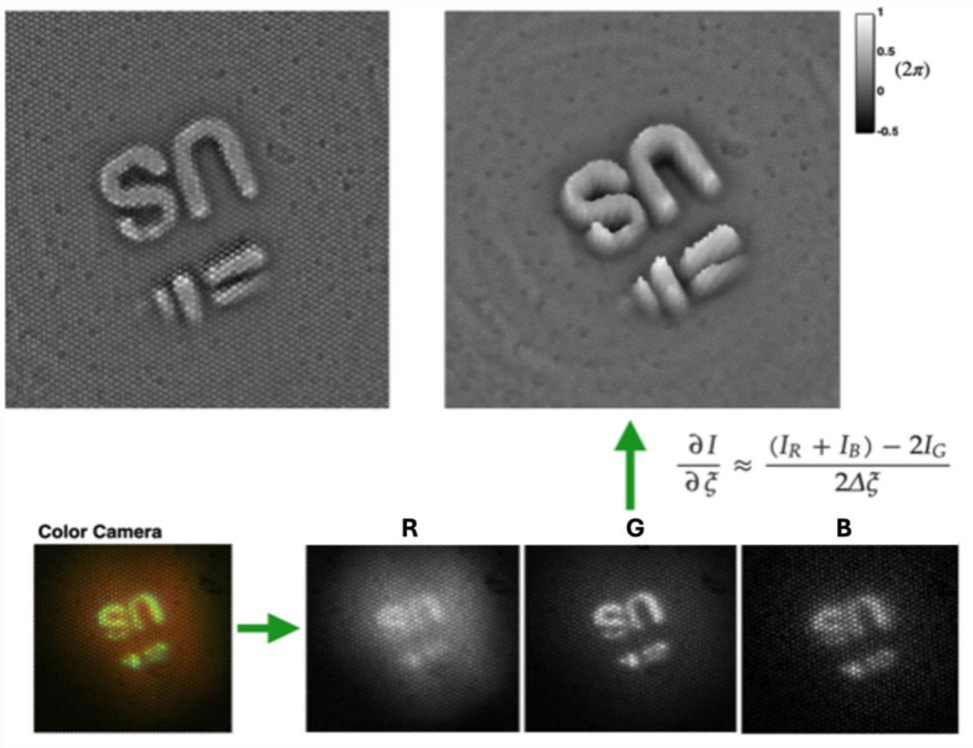

Figure S5. The first proof of concept measurement of phase retrieval from RGB spectral channels was performed using a binary amplitude USAF target images by the metalens through the CFB. The bottom row shows the color image and the corresponding RGB channels of the measurement. The top row shows the retrieved phase in radians performed in the same manner as for the phase objects in the main text. $\xi = \lambda z$ represents the combined independent spectral-focal variable that encodes phase contrast in the intensity derivative. The imaging objective used for the measurement is an achromat, hence encoding the intensity derivative in (IR+IB)-2IG instead of (IR-IG) or (IG-IB).

S7: Scanning Electron Microscope images of Benchmark Phase Calibration Target

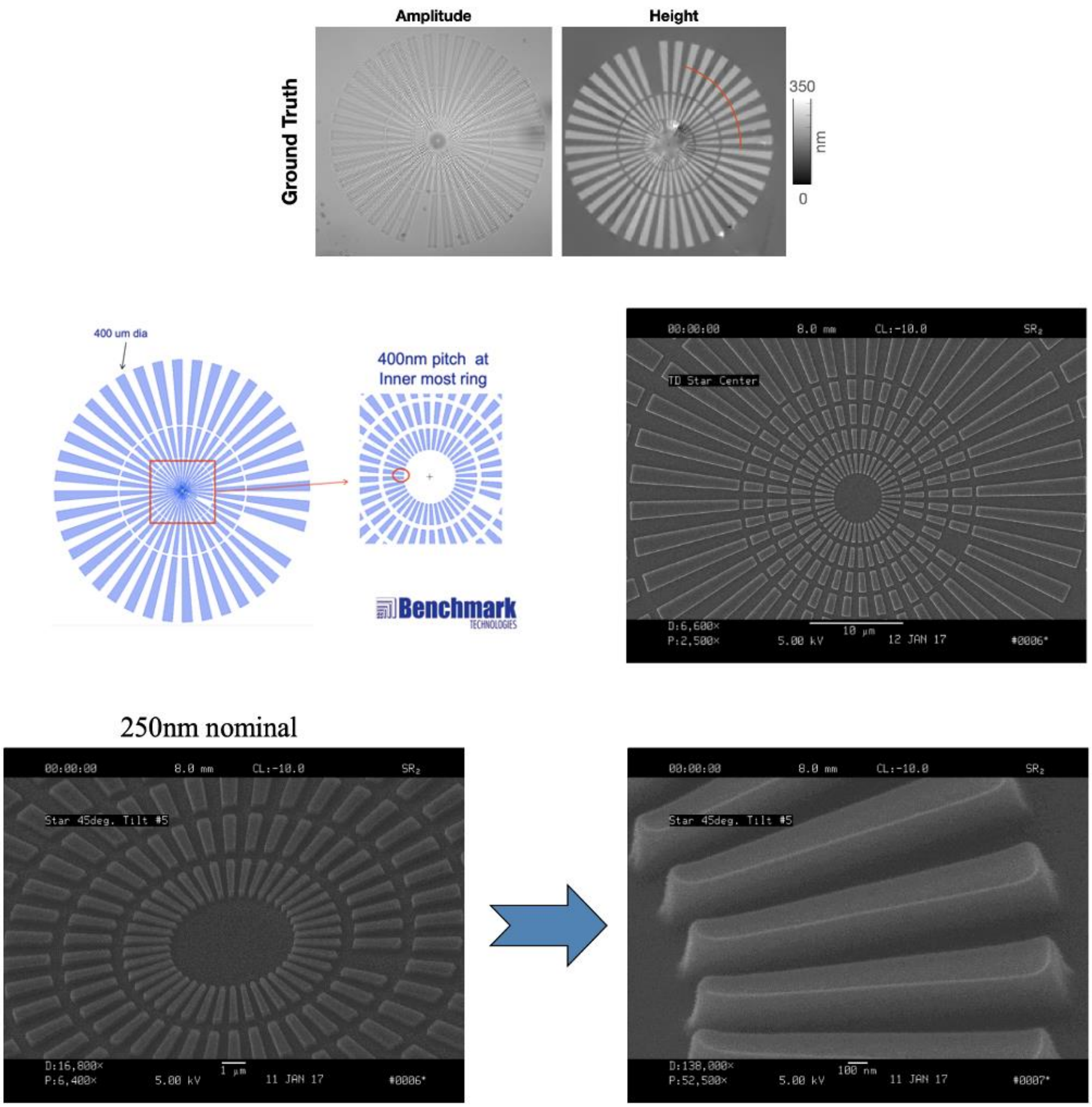

Figure S6. Optical (top row) and Scanning electron microscope (bottom rows) images of the Seimen's star used as ground truth for calibrating our QPI. Optical microscopy is performed with a phasics camera on a widefield transmission Nikon microscope. The 250 nm tall, 400 um wide, phase calibration target is one of a series of targets of varying heights and shapes on glass substrate manufactured by Benchmark Technologies Inc. Images (reproduced with permission).

S8: Seimen's star ground truth validation

Our metalens based QPI is calibrated and validated first with a known phase object. Benchmark Inc., located out of Boulder CO, is one of the first to have a catalog of standardized phase targets for visible light imaging that can be purchased off-the-shelf. The targets include Seimen's stars, concentric cake structures, USAF style numbers and bars etc., each of various diameters and heights ranging from sub 100 nm range to many micrometers . The shapes are etched directly into glass and validated with electron microscope measurements (Fig. S6). We use the calibration target to tune/train our regularization parameter in QPI which is held constant during the experiments.

S9: Phase resolution: minimum height of calibration target resolved by our QPI

The minimum phase resolution of 0.2π achieved by our method is based on the measurement of a 100 nm tall Seimen's star target (Fig. S7) . The blue channel is severely distorted due to higher order aberrations near the phase resolution limit; however, with the TIE, a reasonable value of the quantitative phase is still achieved with the other two spectral channels of the color camera.

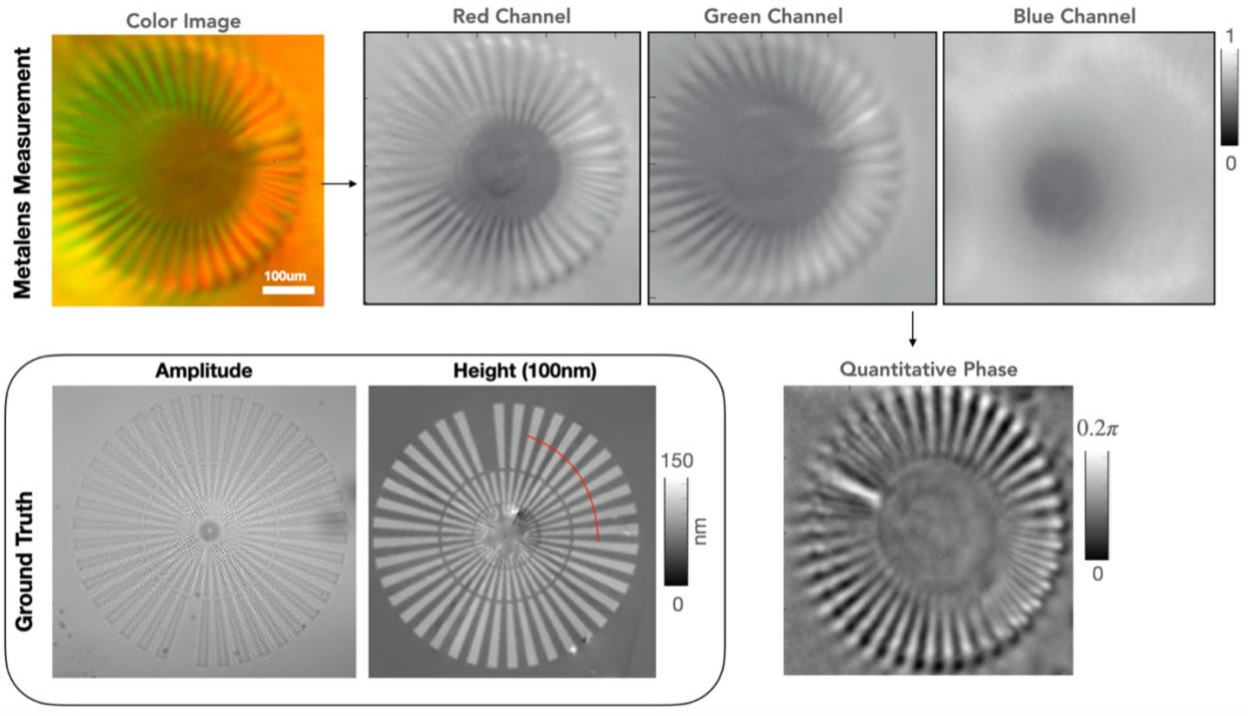

Figure S7. The metalens images of a 100 nm tall Seimen's star target (0.2π phase shift corresponding to a glass substrate with index contrast $n - 1 = 1.52 - 1 = 0.52$, with white light illumination and a CCD color camera allows solving for the phase from just two spectral channels, approaching the lower limit of our QPI's phase resolution.

S10: Choosing the object and image distance for a single lens imaging system.

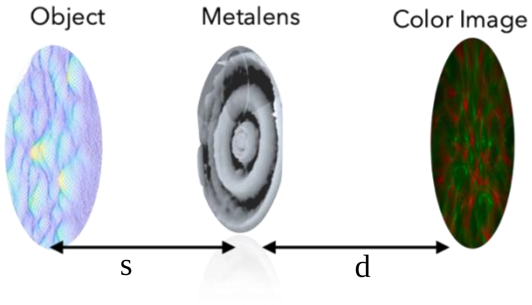

Here we derive how far to place a metalens (with focal length f and radius r) from the object to form a real image, such that we maximize resolution and minimize the imaging system's volume.

Resolution is given by $\delta x_{min} = \frac{\lambda}{\sin\theta} = \frac{\lambda}{r\,/\sqrt{s^2+r^2}} = \lambda\sqrt{1+s^2/r^2}$ where r is the radius of the metalens and θ is half the cone angle subtended by the metalens at the object plane. Hence the smallest possible value of object distance s will be preferred for best resolution while keeping $s>f$.

Imaging system volume is given by $V = \pi r^2(s+d)$ for the enclosing cylinder.

Hence, using the lens law, we write the volume minimization as

$\min_{s,d}\ V = \pi r^2(s+d)$ such that the lens law $\frac{1}{s}+\frac{1}{d}=\frac{1}{f}$ is satisfied.

The lens law can help express the imaging system volume in terms of a single variable s,

$$V = \pi r^2\left(s+\frac{sf}{s-f}\right) = \pi r^2\left(\frac{s^2}{s-f}\right)$$

To minimize, find where the volume function has an extremum by setting the derivative to zero,

$$\frac{dV}{ds} = 0$$

$$\Rightarrow \ \pi r^2\left(\frac{2s}{s-f}\right) - \frac{s^2}{(s-f)^2} = 0$$

$$\Rightarrow \ \pi r^2 \frac{s(s-2f)}{(s-f)^2} = 0 \ \Rightarrow s \ = 2f$$

Hence the minimum volume of the imaging system is achieved at $s = 2f$ and $d = 2f$, corresponding to the "4f" imaging condition with a single lens with unit magnification.

S11: Pseudo Code (Matlab)

We now document the verbatim MATLAB (version R2023a) code to elucidate the numerical methods and algorithms for digital refocusing and transport of intensity-based phase recovery. Signal Processing toolbox may be needed to execute the Fast Fourier Transform (fft) function.

Fresnel Propagator for digital refocusing:

The following code is used to numerically propagate an electric field for digital refocusing. The electric field is a complex valued 2D matrix that has the intensity and phase as the magnitude and argument of the complex valued field.

```
function [Ef,x,y,Fx,Fy,H] = fresnel_prop(E0,ps,lam,z)
%Function Input: Initial field in x-y, wavelength lam, pixel size in um, propagation  distance z
%Function output: Final field in x-y after Fresnel Propagation
% (ref pg 67, J Goodman, Introduction to Fourier Optics)
[M,N] = size(E0);
xsize =  ps*N; ysize = ps*M;
x = linspace(-xsize/2,xsize/2,N);   %Creating the x-y grid
y = linspace(-ysize/2,ysize/2,M);
%Create frequency axis
wx =2*pi*(0:(N-1))/N; %Create unshifted default omega axis
fx = 1/ps*(wx-pi*(1-mod(N,2)/N))/2/pi;
%Shift zero to center - for even case, pull back by pi, for odd case by pi(1-1/N)
wy =2*pi*(0:(M-1))/M; %Create unshifted default omega axis
```

```
fy = 1/ps*(wy-pi*(1-mod(N,2)/N))/2/pi;

%Shift zero to center - for even case, subtract pi, for odd case subtract pi(1-1/N)

[Fx,Fy] = meshgrid(fx,fy);

 %Point spread function h=H(kx,ky)  for propagation

H = exp(1i*2*pi/lam*z)*exp(1i*pi*lam*z*(Fx.^2+Fy.^2));

E0fft = fftshift(fft2(E0));

G = H.*E0fft;                              % Convolution in frequency domain

g = ifft2(ifftshift(G));                    %Output after deshifting the Fourier transform

Ef=g;                                      %Output field; Intensity is Ef^2

end
```

Transport of Intensity phase retrieval:

The phase retrieval algorithm in its most basic form accepts three intensity matrices corresponding to sequential defocus distances or spectral channels. For three colors, the middle color corresponds to the lambda or wavelength input used (green). Epsilons are the regularizers used for our TIE based phase recovery and are held constant after a calibration step given the same light level and experimental setup. For spectral TIE, the same code applies by substituting $dz = -z\frac{d\lambda}{\lambda}$.

```
function [Phi_xy,Psi_xy,Grad_Psi_x,Grad_Psi_y,grad2x,grad2y] =

TIE (IR,IG,IB,ps,lambda,dz,epsilon1,epsilonI)

%Accepts thee intensity matrixes (red, green, blue):  IR, IG,IB

% z distance between intensity planes:      dz =  dλ/λ*z    since λ* z = constant

%Pixel Size :                                 ps (1 um)

%Wavelength :                                 lambda (0.628 um)

%epsilon1   :                                 Normalize when dividing by zero frequency
```

```
%epsilonI   :                                       Normalize when dividing  zero intensity

%Output      :                                      Reconstructed Phase


N = size(IR,1);

k = 2*pi/lambda;

xsize =  ps*(N-1); ysize = ps*(N-1); %square grid with N*N points

epsilon = 1e-7;  %This parameter is experiment dependent


%Create space axes

x = linspace(-xsize/2,xsize/2,N);   %N point sampling over xsize

y = linspace(-ysize/2,ysize/2,N);


%Create frequency axis

wx =2*pi*(0:(N-1))/N;           %Create unshifted default omega axis

%Shift zero to centre - for even case, pull back by pi, for odd case by pi(1-1/N)

fx = 1/ps*(wx-pi*(1-mod(N,2)/N))/(2*pi);

[Fx,Fy] = meshgrid(fx,fx);

% Solve the first Laplacian  for Psi

Del2_Psi_xy = (1*k*((IR+IB/2)-IG)/dz);

Psi_xy = poisson_solve_symm(Del2_Psi_xy,ps,N,epsilon1);

[Grad_Psi_x, Grad_Psi_y] = gradient(Psi_xy/ps); %Take the x and y gradients

%Divide by intensity

Grad_Psi_x = Grad_Psi_x./(IG+epsilonI);Grad_Psi_y = Grad_Psi_y./(IG+epsilonI);
```

```
%Generate the second Laplacian

[grad2x,dummy1] = gradient(Grad_Psi_x/ps);[dummy2,grad2y]=gradient(Grad_Psi_y/ps);

Del2_Phi_xy = grad2x +grad2y;

%Solve the second Laplacian for the Phase

Phi_xy = poisson_solve_symm(Del2_Phi_xy,ps,N,epsilon2);

end
```

Auxiliary function for solving Laplacian (Poisson Solver)

The following function solves the Laplacian $\nabla^2\Psi = func$

```
function Psi_xy = poisson_solve_symm(func,ps,epsilon)

Del2_Psi_xy = func; %Epsilon added to denominator for divide by zero exception

Del2_Psi_uv = fftshift(fft2(Del2_Psi_xy));

Psi_uv = Del2_Psi_uv./(-4*pi^2*(Fx.^2+Fy.^2+epsilon));

Psi_xy = ifft2(ifftshift(Psi_uv),'symmetric');

end
```

Data:

Available upon request from first or last author(s).
